# The genetic diversity of human papillomavirus types from the species *Gammapapillomavirus* 15: HPV135, HPV146, and HPV179

**DOI:** 10.1371/journal.pone.0249829

**Published:** 2021-05-06

**Authors:** Lea Hošnjak, Boštjan J. Kocjan, Branko Pirš, Katja Seme, Mario Poljak

**Affiliations:** 1 Faculty of Medicine, Institute of Microbiology and Immunology, University of Ljubljana, Ljubljana, Slovenia; 2 Dermacenter, Ljubljana, Slovenia; Universidad de Chile, CHILE

## Abstract

**Objectives:**

To determine the prevalence, viral load, tissue tropism, and genetic variability of novel human papillomavirus (HPV) type 179, which is etiologically associated with sporadic cases of common warts in immunocompromised patients, and phylogenetically related HPV types 135 and 146.

**Methods:**

The representative collection of 850 HPV-associated clinical samples (oral/nasopharyngeal/anal, archival specimens of oral/oropharyngeal/conjunctival/cervical/skin cancer, benign lesions of the larynx/conjunctiva/skin, and eyebrows), obtained from immunocompetent individuals, was tested for the presence of HPV179, HPV135, and HPV146 using type-specific real-time PCRs. To assess the genetic diversity of the HPVs investigated in the non-coding long control region (LCR), several highly sensitive nested PCR protocols were developed for each HPV type. The genetic diversity of HPV179 was additionally determined in 12 HPV179 isolates from different anatomical sites of an only immunocompromised patient included in the study.

**Results:**

HPV179, HPV135, and HPV146 were detected in 1.4, 2.0, and 1.5% of the samples tested, respectively, with no preference for cutaneous or mucosal epithelial cells. One (with five single nucleotide polymorphisms; SNPs), four (with one to six SNPs), and four (with one to eight SNPs) genetic variants of HPV179, HPV135, and HPV146, respectively, were identified among eligible samples. HPV179 isolates from the immunocompromised patient exhibited the identical LCR nucleotide sequence, suggesting that HPV179 can cause generalized HPV infections.

**Conclusions:**

HPV179, HPV135, and HPV146 have a mucocutaneous tissue tropism and are associated with sporadic infections in immunocompromised and immunocompetent individuals. Because the majority of mutations were found outside the major functional domains of the respective LCRs, we assume that HPV179, HPV135, and HPV146 genetic variants pathogenically do not differ from their prototypes. In addition, no association was found between specific HPV179, HPV135, and HPV146 genetic variants and anatomical sites of infection and/or specific neoplasms.

## 1. Introduction

Human papillomaviruses (HPVs) are small, non-enveloped DNA viruses that cluster into five different papillomavirus (PV) genera—*Alphapapillomavirus* (*Alpha*-PV), *Betapapillomavirus* (*Beta*-PV), *Gammapapillomavirus* (*Gamma*-PV), *Mupapillomavirus* (*Mu*-PV), and *Nupapillomavirus* (*Nu*-PV)—based on the nucleotide similarities of their L1 genes [1]. *Gamma*-PV is currently the largest PV genus, with 98 officially recognized HPV types (http://www.hpvcenter.se/html/refclones.html; accessed November 26th, 2020) and at least 204 additional complete viral genome sequences that have been determined solely by next-generation sequencing and are therefore not classified by the HPV Reference Center [2]. Moreover, in comparison to *Alpha*-, *Beta*-, *Mu*-, and *Nu*-PV genera including 14, five, three, and one PV species, respectively, *Gamma*-PV is also the most diverse PV genus, with 27 PV species [2].

Even though HPVs are etiologically associated with the development of several benign and malignant neoplasms of the skin and mucosa, the majority of HPV infections are transient and/or do not cause any apparent lesions or disease [1,3]. Accordingly, *Gamma*-PVs have frequently been detected in histologically normal skin [4–10] in several mucosal anatomical sites, including oral, nasal, and cervical mucosa [11–14], and in the mucocutaneous epithelium of the penis and anal canal [15,16]. In addition, their presence has also been demonstrated in *Alpha*-PV-negative anogenital warts [17,18] and cervical intraepithelial lesions [11,19–22]. Moreover, some previous studies proposed a potential active role of *Gamma*-PVs in the development of skin warts [23], premalignant cutaneous lesions [24,25] and head and neck cancer [26].

Recently, we characterized a novel HPV type (HPV179) in a sample of a cutaneous wart obtained from the left cheek of a 64-year-old renal transplant recipient [8]. Based on the relatively high HPV179 viral load (2,463 viral copies/cell) and the absence of the most common wart-associated HPV types, we concluded that HPV179 was most probably etiologically associated with the development of the cutaneous wart [8]. Based on a comparison of its L1 nucleotide sequence with available L1 nucleotide sequences in known sequence repositories, HPV179 was classified into the genus *Gamma*-PV, species *Gamma*-15, with its closest relatives being HPV135 and HPV146. While HPV135 was originally identified in three different swabs of the skin of a 40-year-old immunocompromised patient, after renal transplantation [27], it was latter also found in the oral [12] and nasal cavity [13], swabs of the anogenital warts [15], and vaginal mucosa [6]. Similarly, HPV146 was first detected in the oral cavity [Chen et al. unpublished data] and additionally also in healthy skin [4], nasal cavity [13], swabs of the anogenital warts [15], and vaginal mucosa [6]. Apart of the partial (437 bp) fragment of HPV135 (AF217673), only reference nucleotide sequences of HPV135 (HM999987) and HPV146 (HM999998) are available in the GenBank Database (NCBI, Bethesda, Maryland, https://www.ncbi.nlm.nih.gov/genbank/).

To the best of our knowledge, limited data are available on the clinical relevance and genetic diversity of three HPV types belonging to the species *Gamma-15*. Therefore, in this study we report on the prevalence, tissue tropism, and genetic diversity of the novel HPV type HPV179, and the closely related HPV types HPV135 and HPV146.

## 2. Materials and methods

### 2.1 Representative collection of clinical samples obtained from immunocompetent and immunocompromised individuals

The prevalence and tissue tropism of HPV179 infections were determined by testing a representative collection of benign and malignant cutaneous and mucosal neoplasms associated with HPV, clinically normal cutaneous and mucosal specimens, and eyebrow hair follicles, as previously described [8]. The collection of DNA isolates consisted of 850 clinical specimens previously obtained from the same number of individuals with histologically confirmed diagnoses of oral and oropharyngeal carcinoma (*n* = 50), laryngeal papilloma (*n* = 31), conjunctival papilloma (*n* = 31), conjunctival carcinoma (*n* = 47), cervical carcinoma (*n* = 31), common warts (*n* = 94), cutaneous carcinoma (*n* = 101), and anogenital warts (*n* = 31). In addition, the study included samples from clinically normal oral mucosa (*n* = 144) and the nasopharynx (*n* = 94), anal canal swabs (*n* = 96) of individuals with anogenital neoplasms, predominantly anogenital warts, and eyebrow hair follicles (*n* = 100).

To determine the prevalence and tissue tropism of HPV135 and HPV146 infections, the same sample collection as for the determination of the prevalence of HPV179, with minimal differences in the number of specimens tested from individual anatomical areas, totaling 874 samples, was used.

To evaluate the LCR genetic diversity of HPV179, twelve additional samples, obtained from the single immunocompromised patient, after renal transplantation, in whom the virus was first identified [8], were included in the study. Of the mentioned 12 samples, eight samples were cutaneous (common warts and swabs of the left cheek, right cheek, and neck, and eyebrow hair follicles; [8]), while 4 were mucosal (swabs of the upper and lower gingiva, and left and right buccal mucosa). All mentioned samples were preprocessed as described previously [8]. All mentioned samples were preprocessed as described previously [8]. Additionally, in order to prevent possible tissue cross-contamination during DNA extraction, an extraction control, including only designated buffers, was placed after each fourth tissue sample. All negative extraction controls were subsequently tested using all down-stream HPV detection methods and were determined as HPV-negative.

All clinical specimens were collected from patients with written informed consent in our past or ongoing studies [reviewed in 8]. All studies were performed according to the Helsinki Declaration and approved by the Ethics Committee of the Slovenian Ministry of Health (consent nos. 131/06/07, 45/04/07, 34/11/06, 83/11/09, 174/05/09, 97/11/09, 100/12/09, and 63/10/13). Anonymized archival tissue specimens (only patients’ sex, age, and immune status were available to the researchers) were obtained from the archives of the Institute of Pathology, Faculty of Medicine, University of Ljubljana. In line with Slovenian legislation, no written informed consent is needed when archival samples are used for research purposes. Nevertheless, the institutional review boards of the Institute of Microbiology and Immunology and the Institute of Pathology, Faculty of Medicine, University of Ljubljana, specifically approved all samples for use in this study.

### 2.2 Potential clinical significance of HPV179, HPV135, and HPV146 infections

In order to assess the biological and potential clinical importance of HPV179, HPV135, and HPV146 infections, the representative collection of clinical samples was tested using two highly sensitive type-specific quantitative real-time polymerase chain reactions (RT-PCRs), allowing amplification of HPV179 and HPV135/HPV146, respectively, in combination with the human beta-globin quantitative RT-PCR [28], making possible estimation of the viral loads, as described previously [8]. Although the development of the HPV179 type-specific quantitative RT-PCR was part of our previous study [8], a multiplex HPV135/ HPV146 type-specific quantitative RT-PCR was designed and developed as part of this study. Both sets of primers (HPV135-L1-RT-PCR-F: 5′-ACAACAACAAACAGATGACAACAG-3′ and HPV135-L1-RT-PCR-R: 5′-TATAGGAGGACAAGTGCCTTTTTG-3′, allowing amplification of 153 bp of the HPV135 genome; HPV146-L1-RT-PCR-F: 5′-GGCTTTGTGGTTACCCAACACT-3′ and HPV146-L1-RT-PCR-R: 5′- GGAGTCGATCAGTGCTGGC-3′, allowing amplification of 127 bp of the HPV146 genome) and probes (HPV135-L1-RT-PCR-P: 5′-YAK-AAACCAGTTAGTTATTGTAGGC-BBQ-3′; HPV146-L1-RT-PCR-P: 5′-FAM-TCCACCATCAAAACCAACTCCT-BBQ-3′) were developed based on the nucleotide sequences of L1 genes of individual HPV types, using Primer3Plus (http://primer3plus.com/cgi-bin/dev/primer3plus.cgi), which simultaneously also allows the *in silico* verification of the primers’ propensity to bind to human DNA. The *in silico* specificity of selected primers and probes was additionally verified using the free-of-charge web applications BLAST (http://blast.ncbi.nlm.nih.gov/Blast.cgi) and MFEprimer-2.0 (http://biocompute.bmi.ac.cn/CZlab/MFEprimer-2.0/index.cgi).

The HPV135 and HPV146 PCR amplification protocol was developed according to the manufacturer’s instructions for the LightCycler 480 Probes Master Kit (Roche Diagnostics, Mannheim, Germany) and adjusted according to the predicted length of PCR amplicons, properties of the target nucleotide sequences, and synthesized primers and probes. The reaction mixture (20 μl) contained 100 ng (up to 5 μl) of extracted DNA, 10 μl of 2x LightCycler 480 Probes Master, 0.5 μM of individual primers, 0.2 μM of individual probes, and sterile deionized water.

The RT-PCR was performed using the LightCycler 480 II RT-PCR Instrument (Roche Diagnostics). Initially, a 10 min denaturation was performed at 95°C, followed by amplification of targets by 40 repetitions of a temperature cycle, consisting of three incubations: 10 s at 95°C, 30 s at 60°C, and 1 s at 72°C. The amplifications of HPV135 and HPV146 were monitored by measuring the fluorescence at 510 and 580 nm, respectively. The amplification was followed by a 30 s cooling of the reaction mixture to 40°C.

The analytical sensitivity of the multiplex RT-PCR assay was determined using triplicates of 10-fold serially diluted plasmids, containing target HPV135 and HPV146 genes, respectively, in concentrations of 1 to 10^9^ copies of viral DNA/reaction, in the presence of 100 ng of human DNA. The correlation coefficients of the standard amplification curves of HPV135 and HPV146, through at least eight orders of magnitude, were evaluated at *R*^2^ = 0.999, respectively. Both amplification efficiencies were also excellent, estimated at 100 and 97.4% for HPV135 and HPV146, respectively. The 100% analytical sensitivity of the HPV135/HPV146 multiplex RT-PCR was estimated at 10 copies of HPV135 or HPV146/reaction.

The analytical specificity of each of the singleplex RT-PCRs, together forming the HPV135/HPV146 multiplex RT-PCR, was verified by testing the complete series of diluted plasmids, containing the phylogenetically most related HPVs (HPV179/HPV135/HPV149), and by determination of the nucleotide sequences of all RT-PCR amplicons, using Sanger sequencing, as described below. No cross-reactivity between individual RT-PCRs was observed.

In addition, in order to analyze the amplification efficiencies of HPV135 and HPV146 in samples with simultaneous infections with both viruses, 500 copies of HPV135/HPV146 per reaction were amplified in the presence of 1 × 10^9^, 1 × 10^8^, 1 × 10^7^, 100, and 10 copies of HPV146/HPV135 per reaction. Using this approach, it was shown that in cases with simultaneous infections with HPV135 and HPV146 both HPV types could be detected using our multiplex RT-PCR, regardless of whether the concentration of one HPV type is significantly higher than the concentration of the other HPV type.

### 2.3 Identification of concomitant HPV infections in HPV179-, HPV135-, and HPV146-positive samples

The presence of concomitant HPV infections in selected HPV179-, HPV135-, and HPV146-positive samples was determined using several in-house and commercially available PCR protocols. Typical causative agents of common warts from the *Alpha*- (HPV types 2, 3, 6, 7, 10, 11, 13, 27, 28, 29, 32, 40, 42, 43, 44, 57, 74, 77, 78, 91, 94, 117, and 125), *Mu*- (HPV types 1 and 63), and *Nu*-PV (HPV41) genera were investigated using three sets of broad-spectrum primers, HVP2/B5, low-risk *Alpha*-PV, and CPI/CPIIg, as described previously [29–31]. The presence of *Gamma*-PVs in clinical specimens of common warts and nasopharyngeal swabs was determined using the previously described broad-spectrum *Gamma*-PV-E1F/E1R primers, according to the previously published protocol [32]. All products of conventional PCRs were further evaluated using agarose gel electrophoresis and Sanger sequencing of both DNA strands on an ABI3500 Genetic Analyzer (Applied BioSystems, Life Technologies, Carlsbad, CA). The nucleotide sequences obtained were processed with Vector NTI Advance v11 software (Invitrogen, Carlsbad, CA) and compared with nucleotide sequences available in the GenBank database, making possible determination of the HPV types present [30].

In clinical samples from the anogenital area (anogenital warts and swabs of the anal canal), the commercially available Linear Array HPV Genotyping Test (Roche Diagnostics) was used to detect the presence of the 37 clinically most important *Alpha*-PVs (HPV types 6, 11, 16, 18, 26, 31, 33, 35, 39, 40, 42, 44, 45, 51, 52, 53, 54, 56, 58, 59, 61, 62, 64, 66, 67, 68, 69, 70, 71, 72, 73, 82, 81, 82, 83, 84, and 89). In addition, the commercially available RHA Kit Skin (beta) HPV (Diassay BV, Rijswijk, Netherlands), allowing detection of 25 different *Beta*-PV types (HPV types 5, 8, 9, 12, 14, 15, 17, 19, 20, 21, 22, 23, 24, 25, 36, 37, 38, 47, 49, 75, 76, 80, 92, 93, and 96), was used in samples of common warts, cutaneous and conjunctival cancer, anogenital warts, swabs of the oral cavity, nasopharynx and anal canal, and eyebrow hair follicles. Both tests were performed according to the manufacturers’ instructions.

### 2.4 Long control region genetic diversity of HPV179, HPV135, and HPV146

In order to assess the genetic diversity of HPV179, HPV135, and HPV146 in the non-coding genomic long control region (LCR), several highly sensitive nested PCR protocols, adjusted for use with archival tissue specimens, were developed for each HPV type, using Vector NTI Advance v11 software (Invitrogen). Four sets of PCR primers were designed for amplification of the HPV179 LCR, allowing amplification of the complete (outer PCR; HPV179-135-LCR-F: 5′-ATCAGATTGGAATGTTAAATGG-3′ and HPV179-LCR-R: 5′-TTCATTTAGACTGCGTGGC-3′, length of the PCR product: 600 bp) and individual parts of the target region (inner PCRs; HPV179-135-LCR-F and HPV179-LCR-Ri3: 5′-TTTGTACCAGAGTAGGTTGTAAA-3′, length of the PCR product: 300 bp; HPV179-135-LCR-Fi3: 5′-AAACATTTTCAAAGTAAAGACG-3′ and HPV179-LCR-Ri2: 5′-ACATCTATGATTGTTGCCAAC-3′, length of the PCR product: 270 bp; HPV179-LCR-Fi2: 5′-AGCTGTTTTGTACCGCTTAC-3′ and HPV179-LCR-R, length of the PCR product: 160 bp). Similarly as for amplification of the HPV179 LCR, three sets of PCR primers were designed for the amplification of HPV135 and 146 LCRs, respectively, allowing amplification of the complete (outer PCRs; HPV179-135-LCR-F and HPV135-LCR-R: 5′-TCTACAGAGTAATAATAGTTGCCA-3′, length of the PCR product: 600 bp; HPV146-LCR-F: 5′-AAATTGGTATGTTGAATGGC-3′ and HPV146-LCR-R: 5′-CGGAAAATAAGCAGCCAT-3′, length of the PCR product: 600 bp), and individual parts of the target regions (inner PCRs; HPV179-135-LCR-F and HPV135-LCR-Ri: 5′-CTTGAAGGATATGTTCCAATG-3′, length of the PCR product: 300 bp; HPV135-LCR-Fi: 5′-CCGTGAGTATCTTGCCAGC-3′ and HPV135-LCR-R, length of the PCR product: 300 bp; HPV146-LCR-F and HPV146-LCR-Ri: 5′-TGTCCCTGTCTGAACTTGAG-3′, length of the PCR product: 300 bp; HPV146-LCR-Fi: 5′-GCCAACTGGACACTGTTCA-3′ and HPV146-LCR-R, length of the PCR product: 300 bp). The *in silico* specificity of the selected primers was verified using the BLAST and MFEexample-2.0 web applications.

Individual HPV179, HPV135, and HPV146 LCR amplification protocols were developed following the manufacturer’s instructions for the FastStart High Fidelity PCR System (Roche Diagnostics) and adjusted according to the predicted length of PCR amplicons, properties of the target nucleotide sequences, and synthesized primers. The reaction mixtures (25 μl) of all PCR protocols contained up to 5 μl (100 ng) of extracted DNA, 2.5 μl of the 10x FastStart High Fidelity Reaction Buffer (containing 18 mM MgCl_2_), 200 μM of dNTPs, 0.5 μM of individual primers, and sterile deionized water.

All LCR PCR protocols were performed on a Veriti Thermal Cycler (Applied Biosystems, Life Technologies). Outer HPV179/135/146 LCR PCRs were performed according to the following protocol: a 2 min denaturation at 95°C, followed by amplification of targets by 40 repetitions of a temperature cycle, consisting of three incubations: 30 s at 95°C, 30 s at 54°C, and 1 min at 72°C, a 7 min incubation at 72°C, and final cooling of the reaction mixture to 8°C. Three μl of each of the outer PCR reactions were subsequently used for individual inner PCRs, which were performed under the same temperature conditions as outer PCRs, except for the number of repetitions and annealing temperature, which were lowered to 35 and 52°C, respectively.

The PCR products obtained were further evaluated using agarose gel electrophoresis and Sanger sequencing, as described above. In addition, the presence of single nucleotide polymorphisms (SNPs), insertions, and deletions was assigned based on comparisons of the HPV179/HPV135/HPV146 LCR nucleotide sequences obtained with appropriate reference genomes (HPV179 = HG421739, HPV135 = HM999987, HPV146 = HM999998); a novel viral variant was identified when the query and reference nucleotide sequence differed by at least one nucleotide position. All LCR viral variant nucleotide sequences, obtained in our study, were additionally submitted to the GenBank database.

The analytical sensitivity of all individual PCR reactions—which was verified by testing 10-fold serially diluted plasmids, containing the target HPV179/HPV135/HPV146 genomic regions, in concentrations of 1 to 10^9^ copies of viral DNA/reaction, in the presence of 100 ng of human DNA—was estimated to at least 10 viral copies/reaction.

## 3. Results

### 3.1 Determination of the prevalence, viral load, and tissue tropism of HPV179

Based on the HPV179 RT-PCR testing of a representative collection of various clinical samples (*n* = 850) obtained from immunocompetent individuals, altogether 12 independent HPV179 isolates, seven in our previous study [8] and five in this study, were identified, as shown in Table 1. Specifically, four HPV179-positive isolates were detected among samples from clinically normal oral mucosa, one in a nasopharyngeal sample, four in samples of common warts, one in eyebrow hair follicles, and two among samples of skin cancer. As shown in Table 1, the HPV179 viral load was low in all clinical samples tested and ranged from 1 to 1,400 viral copies/10^4^ cells. In addition, in all HPV179-positive specimens, a number of concomitant HPV infections were demonstrated, most often with *Beta*-PV types. Moreover, in all four HPV179-positive common warts, the presence of well-known etiological agents of these lesions (HPV types 1, 2, and 4) was additionally identified (Table 1).

**Table 1:** Prevalence and tissue tropism of human papillomavirus type 179 (HPV179) in immunocompetent individuals.

Tissue type	Anatomical location (sample type)	Histology/clinical picture	No. of positives/samples tested (prevalence in %)	Sample no.	Viral load (viral copies/10^4^ cells)	Infections with other HPVs
**Mucosal**	Oral cavity (swab)	Normal clinical picture^a^	4/144 (2.8)	Sm-117	nd	159
				Sm-121	nd	nd
				Sm-157	nd	159
				Sm-158	nd	nd
	Oral cavity and oropharynx (FFPE)^a^	SCC	0/50	–	–	–
	Nasopharynx (swab)	Normal clinical picture^a^	1/94 (1.1)	bp-1520	nd	12^c^
	Larynx (fresh tissue)^b^	Papilloma	0/31	–	–	–
	Conjunctiva (FFPE)^b^	Papilloma	0/31	–	–	–
	Conjunctiva (FFPE)	SCC	0/47	–	–	–
	Cervix (FFPE)^a^	SCC	0/31	–	–	–
**Cutaneous**	Skin (FFPE, fresh tissue)^a^	Common warts	4/94 (4.2)	P00061	2	1, **2**, 5, 36
				P00073	1.2	**2**, **4**, 5, 9, 12, 17, 24, 93
				ZU-1	1.2	**2**, 12, 24, 76, 93
				ZU-10	11	**4**
	Eyebrows (hair follicles)^a^	–	1/100 (1.0)	3380-o-pool	91	22, 38
	Skin (FFPE)^a^	SCC	1/50 (2.0)	P00225	1,400	9, 21, 24, 36, 151
	Skin (FFPE)^a^	BCC	1/51 (2.0)	P00268	32	9, 17, 23, 24, 38, 92, 92
	Anogenital area (fresh tissue)^a^	Anogenital warts	0/31	–	–	–
**Muco-cutaneous**	Anal canal (swab)	Anogenital neoplasms	0/96	–	–	–
**Total**			**12/850 (1.4)**			

SCC = squamous cell carcinoma; BCC = basal cell carcinoma; FFPE = formalin-fixed and paraffin-embedded samples; nd = not done

^a^refers to the clinically healthy tissue, as observed by the clinicians upon the examination of patients and swab sampling–as only swabs were available for these anatomic sites, their histological evaluation could not be performed

^b^previously published in Hošnjak et al. 2015 [8]; the HPV179 viral load was determined only in tissue specimens and eyebrow hair follicles

^c^the sample was not tested for the presence of *Gamma*-PVs other than HPV179; the most probable etiological agents of benign skin neoplasms are marked in red boldface.

### 3.2 Determination of the prevalence, viral load, and tissue tropism of HPV135

As shown in Table 2, based on the HPV135 RT-PCR testing of a representative collection of various clinical samples (*n* = 874) obtained from immunocompetent individuals, 17 independent HPV135 isolates were identified, originating from the oral cavity (*n* = 1), nasopharynx (*n* = 2), conjunctival squamous cell carcinoma (SCC) (*n* = 2), common warts (*n* = 4), eyebrows (*n* = 5), and anal canal (*n* = 3). The HPV135 viral load was low in all clinical samples tested and ranged from five to 9,900 viral copies/10^4^ cells (Table 2). In addition, concomitant viral infections, with *Alpha*- and *Beta*-PVs, were demonstrated in HPV135-positive samples from the anal canal. Moreover, the presence of HPV135 was also demonstrated in both common warts (sample nos. P00080 and P00081), which were most probably caused by infections with HPV179 and HPV184, respectively [8]. Whereas in the third HPV135-positive common wart (sample no. P00073) the presence of HPV2 and HPV4 was additionally demonstrated, no common viral etiological agents were identified in the fourth HPV135-positive common wart (sample no. ZU-9) (Table 2).

**Table 2:** Prevalence and tissue tropism of human papillomavirus type 135 (HPV135) in immunocompetent individuals.

Tissue type	Anatomical location (sample type)	Histology/clinical picture	No. of positives/samples tested (prevalence in %)	Sample no.	Viral load (viral copies/10^4^ cells)	Infections with other HPVs
**Mucosal**	Oral cavity (swab)	Normal clinical picture^a^	1/153 (0.7)	Sm-97	nd	nd
	Oral cavity and oropharynx (FFPE)	SCC	0/40	–	–	–
	Nasopharynx (swab)	Normal clinical picture^a^	2/104 (1.9)	bp-1356	nd	14^b^
				bp-1893	nd	4^c^
	Larynx (fresh tissue)	Papilloma	0/31	–	–	–
	Conjunctiva (FFPE)	Papilloma	0/31	–	–	–
	Conjunctiva (FFPE)	SCC	2/51 (3.9)	X8	72	20, 21
				Z18	9,900	nd
	Cervix (FFPE)	SCC	0/50	–	–	–
**Cutaneous**	Skin (FFPE, fresh tissue)	Common warts	4/89 (4.5)	P00080	62	8, 9, 23, 49, 150, 174, 179, **184**
				P00081	150	8, 9, 12, 14, 15, 20, 23, 24, 49, 75, 80, 93, 150, 174, **179**, 184
				ZU-9	539	124
				P00073	5	**2**, **4**, 5, 9,12, 17, 24, 93
	Eyebrows (hair follicles)	–	5/102 (4.9)	061-3-Do	90	nd
				K034-2-Do	576	nd
				K022-Do	17	nd
				031-7-do	29	nd
				K001-3-do	33	nd
	Skin (FFPE)	SCC	0/45	–	–	–
	Skin (FFPE)	BCC	0/48	–	–	–
	Anogenital area (fresh tissue)	Anogenital warts	0/30	–	–	–
**Muco-cutaneous**	Anal canal (swab)	Anogenital neoplasms	3/100 (3.0)	A347	nd	6^b^
				A361	nd	59, 12
				A451	nd	61, 62, 66, 72^b^
**Total**	** **		**17/874 (2.2)**	** **	** **	** **

SCC = squamous cell carcinoma; BCC = basal cell carcinoma; FFPE = formalin-fixed and paraffin-embedded samples; nd = not done; the HPV135 viral load was determined only in tissue specimens and eyebrow hair follicles

^a^refers to the clinically healthy tissue, as observed by the clinicians upon the examination of patients and swab sampling–as only swabs were available for these anatomic sites, their histological evaluation could not be performed

^b^the sample was not tested for the presence of *Gamma*-PVs other than HPV179

^c^the samples were not tested for the presence of *Beta*-PVs; the most probable etiological agents of benign skin neoplasms are marked in red boldface.

### 3.3 Determination of the prevalence, viral load, and tissue tropism of HPV146

Through HPV146 RT-PCR testing of a representative collection of various clinical samples (*n* = 874) obtained from immunocompetent individuals, 13 independent HPV146 isolates were detected (Table 3). Specifically, three HPV146-positive isolates were identified in oral swab samples, one in common warts, two in eyebrow hair follicles, one in skin carcinoma, one in anogenital warts, and five in anal canal swab samples. As shown in Table 3, the HPV146 viral load in the common wart tissue sample (P00146) was estimated at 1,134 viral copies/10^4^ cells. In addition, the HPV146 viral load was also low (247–460 viral copies/10^4^ cells) in the other clinical samples tested, with the exception of the anogenital wart (sample no. P01362), in which it was estimated at approximately two copies/cell. That tissue sample also contained HPV6, an *Alpha*-PV type, which is most commonly associated with the development of anogenital warts (Table 3).

**Table 3:** Prevalence and tissue tropism of human papillomavirus type 146 (HPV146) in immunocompetent individuals.

Tissue type	Anatomical location (sample type)	Histology/clinical picture	No. of positives/samples tested (prevalence in %)	Sample no.	Viral load (viral copies/10^4^ cells)	Infections with other HPVs
**Mucosal**	Oral cavity (swab)	Normal clinical picture^a^	3/153 (2.0)	Sm-124	nd	159
				Sm-125	nd	nd
				Sm-135	nd	nd
	Oral cavity and oropharynx (FFPE)	SCC	0/40	–	–	–
	Nasopharynx (swab)	Normal clinical picture^a^	0/104	–	–	–
	Larynx (fresh tissue)	Papilloma	0/31	–	–	–
	Conjunctiva (FFPE)	Papilloma	0/31	–	–	–
	Conjunctiva (FFPE)	SCC	0/51	–	–	–
	Cervix (FFPE)	SCC	0/50	–	–	–
**Cutaneous**	Skin (FFPE, fresh tissue)	Common warts	1/89 (1.1)	P00146	1,134	b,c
	Eyebrows (hair follicles)	–	2/102 (2.0)	K034-2-Do	460	nd
				025-8-do	247	nd
	Skin (FFPE)	SCC	1/45 (2.2)	P00236	1,146	nd
	Skin (FFPE)	BCC	0/48	–	–	–
	Anogenital area (fresh tissue)	Anogenital warts	1/30 (3.3)	P01362	16,921	**6**^b^
**Muco-cutaneous**	Anal canal (swab)	Anogenital neoplasms	5/100 (5.0)	A350	nd	6^b^
				A387	nd	11, 51, 73^b^
				A426	nd	6^b^
				A435	nd	6, 59^b^
				A475	nd	6, 14, 16, 21, 23, 42, 53, 59, 61, 89, 93
**Total**	** **		**13/874 (1.5)**	** **	** **	** **

SCC = squamous cell carcinoma; BCC = basal cell carcinoma; FFPE = formalin-fixed and paraffin-embedded samples; nd = not done; the HPV146 viral load was determined only in tissue specimens and eyebrow hair follicles

^a^refers to the clinically healthy tissue, as observed by the clinicians upon the examination of patients and swab sampling–as only swabs were available for these anatomic sites, their histological evaluation could not be performed

^b^samples were not tested for the presence of *Beta*-PVs

^c^the sample was not tested for the presence of *Gamma*-PVs other than HPV179; the most probable etiological agents of benign skin neoplasms are marked in red boldface.

### 3.4 Viral variants of HPV179, HPV135, and HPV146

**Fig 1:**
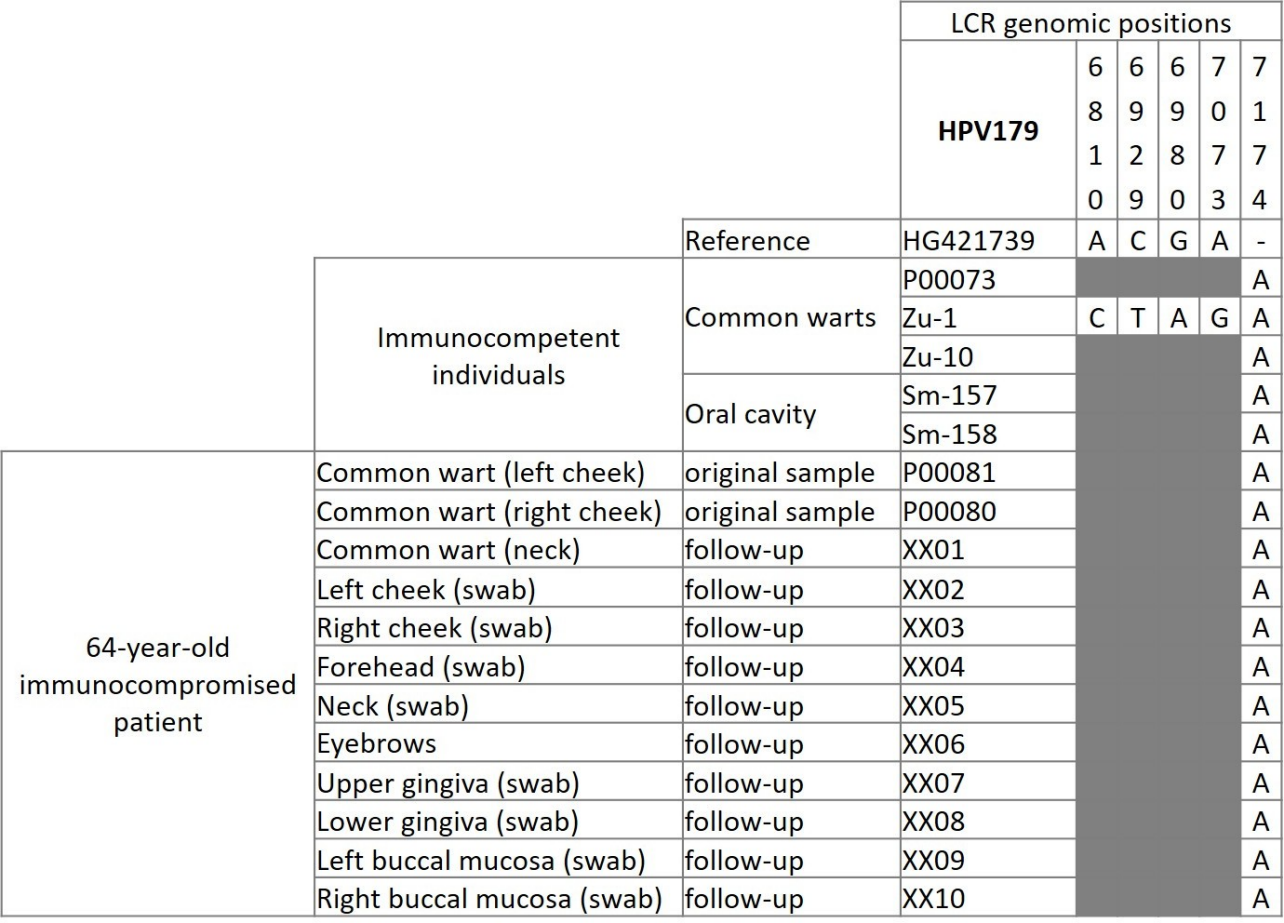
Long control region viral variants of human papillomavirus type 179 in 12 samples obtained from an immunocompromised patient and five independent samples obtained from immunocompetent individuals (GenBank acc. nos. MW574219 –MW574235). The top of the frequency table shows the nucleotide sites of substitutions/insertions in the HPV179 isolates investigated according to the HPV179 reference genome. Nucleotide sites where no substitutions or deletions were observed are marked gray.

After excluding one sample with insufficient remaining DNA (Table 1, sample no. bp-1520) and six samples with low viral DNA concentrations (Table 1, sample nos. Sm-117, Sm-121, P00061, P00225, P00268, and 3380-o-pool), 17 HPV179 isolates (GenBank accession numbers MW574219 –MW574235) were used to identify its LCR genetic variants, of which 12 isolates belonged to the same immunocompromised patient, in which the virus was initially identified [8], and five isolates were obtained from independent immunocompetent individuals (Table 1). As shown in Fig 1, no differences were observed within the HPV179 LCR genetic variants of the initial immunocompromised patient. In addition, among the other five HPV179 isolates, only one viral variant, which differed from the reference nucleotide sequence at five nucleotide sites (5/505; 0.99%), was detected in a sample of a common wart obtained from an immunocompetent individual (sample no. Zu-1; Table 1). Interestingly, all nucleotide sequences of the LCR genomic region obtained from clinical samples differed from the reference nucleotide sequence by one insertion (A) at nucleotide site 7,174 (Fig 1).

**Fig 2:**
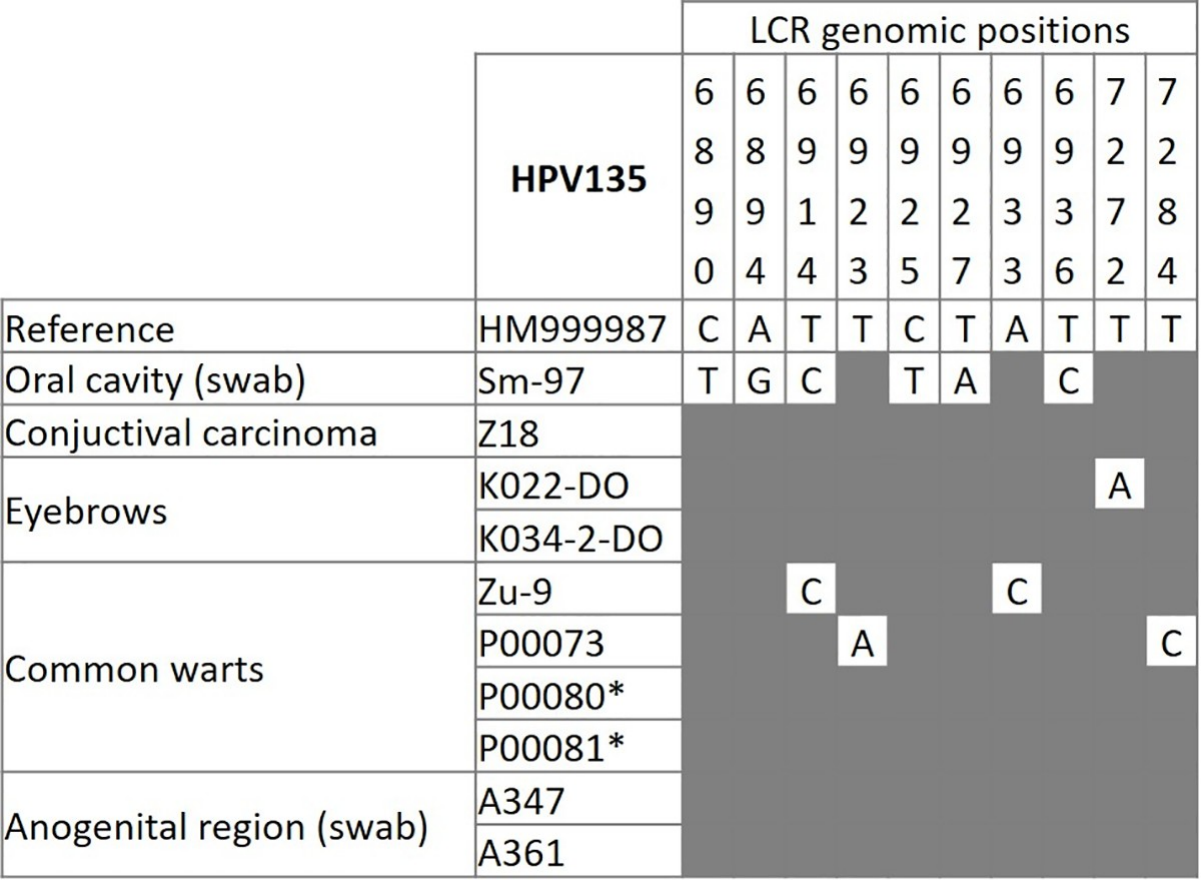
Long control region viral variants of human papillomavirus type 135 in two samples obtained from an immunocompromised patient (P00080 and P00081, marked with a star) and in eight independent samples obtained from immunocompetent individuals (GenBank acc. nos. MW574236 –MW574245). The top of the frequency table shows the nucleotide sites of substitutions in HPV135 isolates investigated according to the HPV135 reference genome. Nucleotide sites where no substitutions or deletions were observed are marked gray.

Altogether 10 different HPV135 isolates (GenBank acc. nos. MW574236 –MW574245), originating from the same immunocompromised patient (sample nos. P00080 and P00081, Table 2 and Fig 2) and five independent immunocompetent individuals, were included in the analysis of LCR genetic variants. Unfortunately, two samples with insufficient residual DNA (Table 2, sample nos. Bp-1356 and A451) and five samples with low viral DNA concentrations (Table 2, sample nos. Bp-1893, X8, 061-3-do, 031-7-do and K001-3-do) had to be excluded from these analyses. As shown in Fig 2, among ten HPV135 isolates, four LCR genetic variants were identified, which differed from the reference nucleotide sequence at one (1/423; 0.24%) to six nucleotides (6/423; 1.42%). Specifically, HPV135 viral variants were identified in oral swab samples, eyebrow hair follicles, and common warts of immunocompetent individuals (Fig 2).

**Fig 3:**
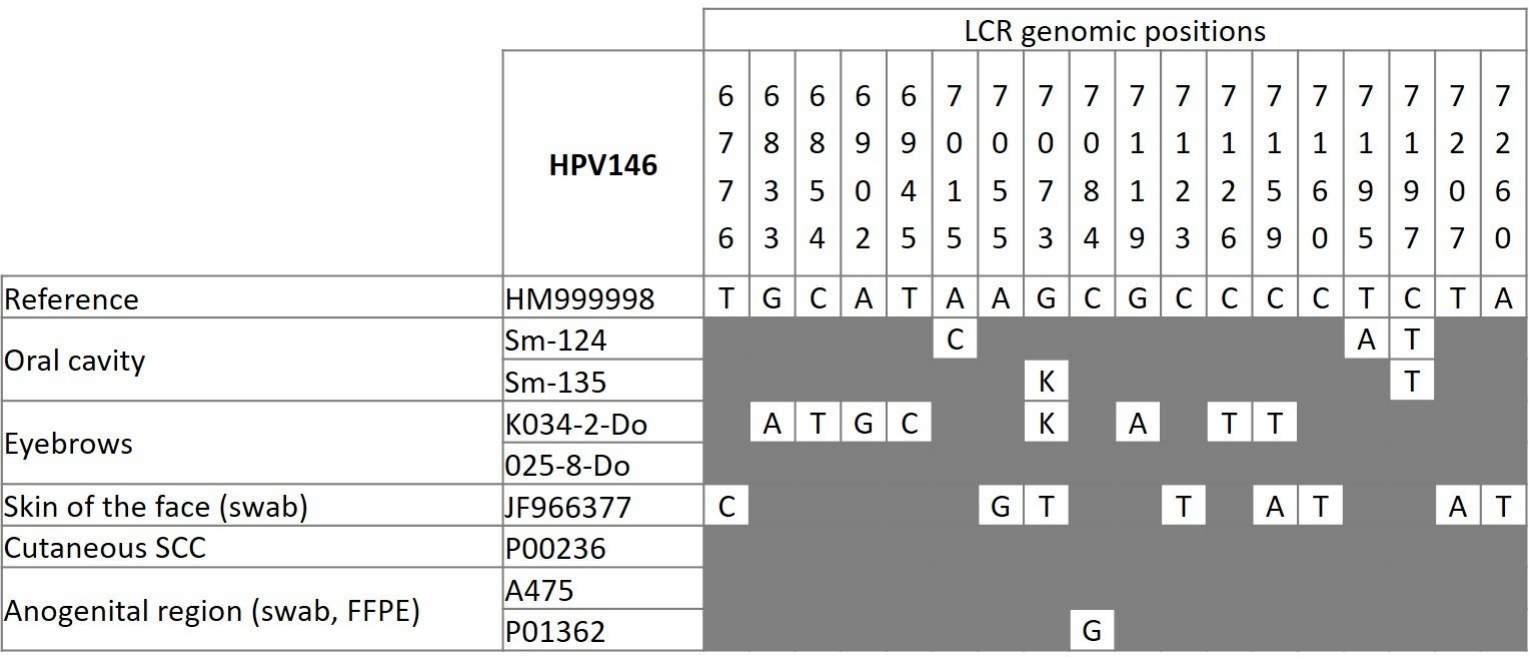
Long control region viral variants of human papillomavirus type 146 in eight independent samples obtained from immunocompetent individuals (GenBank acc. nos. MW574246 –MW574252 and JF966377). The top of the frequency table shows the nucleotide sites of substitutions in HPV146 isolates investigated according to the HPV146 reference genome. Nucleotide sites where no substitutions or deletions were observed are marked gray. *The viral variants consist of at least two variants of the LCR genomic region; degenerate bases (wobble) by IUB: K (G/T).

After excluding six HPV146-positive samples with low viral DNA concentrations (Table 3, sample nos. Sm-125, P00146, A350, A387, A426, A435), seven isolates obtained from our collection of clinical samples (GenBank acc. nos. MW574246 –MW574252) and one HPV146 isolate from the GenBank database (acc. no. JF966377) were included in analysis of HPV146 LCR genetic variants. Of the eight HPV146 isolates, five LCR genetic variants were identified: two in the swabs of the oral cavity, one in eyebrow hair follicles, one in the facial skin swab, and one in the anogenital region (Fig 3). As shown in Fig 3, HPV146 LCR genetic variants differed from the reference nucleotide sequence at one (1/509; 0.20%) to eight nucleotides (8/509; 1.57%). Most probably simultaneous infection with two HPV146 viral variants was demonstrated in two clinical samples (sample nos. Sm-135 and K034-2-do).

## 4. Discussion

*Gamma*-PVs have an extremely broad tissue tropism, infecting both cutaneous as well as mucosal and mucocutaneous epithelium, and have so far been identified in various clinical samples, including benign and malignant cutaneous neoplasms, and clinically normal skin, the oral cavity, the nasopharnx, and the anogenital area (penis, cervix, and anal canal) [7,16,26,33,34]. Similarly, the presence of the phylogenetically closest HPV179 relatives (HPV135 and HPV146) has been detected in several samples from the oral and nasal cavities, vaginal mucosa, anogenital warts, and histologically normal skin [4,6,12,13,15,27, Chen et al. unpublished data].

In order to accurately determine the tissue tropism and potential clinical significance of *Gamma*-15 PVs, the previously described HPV179 type-specific quantitative RT-PCR [8] and newly designed and developed highly sensitive and specific multiplex HPV135/HPV146 quantitative RT-PCR were used in this study. Both tests were applied to a collection of clinical samples compiled to contain the most important HPV-related cancerous (SCC and basal cell carcinoma (BCC) of the skin, SCC of the oral cavity and oropharynx, conjunctiva, and cervix) and benign (papillomas of the larynx and conjunctiva, and common and anogenital warts) neoplastic changes of the skin and mucosa. In addition, swabs of the clinically normal oral cavity and nasopharynx, and eyebrow hair follicles, and anal canal swabs were also included to evaluate the presence of *Gamma*-15 PVs in healthy individuals.

HPV179 was originally identified in a common wart of an immunocompromised patient and, based on the high viral load and absence of the most common etiological factors of common warts (HPV2, 27, 57) [35], it was characterized as its most probable causative agent [8]. Subsequently, seven additional HPV179-isolates, four from common warts (4/94; 4.2%) and single isolates from eyebrow hair follicles (1/100; 1.0%), cutaneous SCC (1/50; 2.0%), and BCC (1/51; 1.96%), were detected in our previous study [8]. In this study, which mostly focused on mucosal HPV179 infections, HPV179 was additionally identified in four samples from clinically normal oral mucosa (4/144; 2.8%) and in one sample from clinically normal nasopharyngeal mucosa (1/94; 1.1%). Based on the extremely low HPV179 viral load in all clinical samples tested, it could be proposed that HPV179, at least in immunocompetent individuals, mostly causes clinically insignificant viral infections of the skin and mucosa, with no or minimal production of mature viral particles. Moreover, in almost all HPV179-positive clinical specimens, concomitant HPV infections with up to eight *Beta*-PVs were demonstrated, confirming the results of previously published studies, suggesting that these viruses can infect cutaneous and mucosal epithelia of different anatomical sites, similarly to *Gamma*-PVs [3,6,12,13,15,28,31,36,37].

Testing of a representative collection of samples (17/874; 2.2%) confirmed the results of previous studies, in which HPV135 was found in the oral and nasal cavity and clinically normal skin [4,12,13,15,27]. Furthermore, for the first time HPV135 was also detected in samples of conjunctival SCC, common warts, and the anal canal. Similarly to HPV179, the presence of one to 14 different *Beta*-PVs was identified in most of the HPV135-positive samples, confirming the previous findings that these two PV genera are not tissue-specific [3,6,12,13,15,28,31,36,37].

HPV135 infection has been demonstrated in four cases of common warts, at concentrations of five to 539 viral copies/10^4^ cells that are much lower than previously suggested for viruses that are etiologically associated with the development of such lesions [35,38]. Interestingly, HPV135 has been detected in both skin warts undoubtedly caused by HPV179 and 184, respectively [8]. Due to the presence of HPV types 2 and 4, which are well-known etiological agents of common warts [3], HPV135 was most probably also not involved in the development of the lesion with this combination of HPV types. In the last sample of HPV135-positive common warts, no commonly known causative agents were identified, suggesting that it was most probably caused by the hitherto unknown HPV type. Nevertheless, it should be noted that the overall prevalence of HPV2, HPV4, HPV27, and HPV57 in common warts has been extensively studied in the past, in contrast to viruses, which were the primary focus of our study. Therefore, further studies, on a larger number of samples and including newly identified HPVs, should performed in the future to undoubtedly determine the etiological agents in common warts in which the cause for the development of the mentioned lesions is not transparent.

The HPV135 viral load was also low (17 to 9,900 viral copies/10^4^ cells) in other clinical samples tested, suggesting that in most immunocompetent individuals HPV135 causes clinically insignificant viral infections of the skin and mucosa.

HPV135 has also been detected in anal swabs of men who have sex with men, in addition to the presence of *Alpha*-PVs, confirming the recent findings of our research group and others that the mucocutaneous epithelium of the anal canal is able to host HPVs from different genera [15,28,39].

In our study, HPV135 infection was additionally identified in two samples of conjunctival SCC. To the best of our knowledge, this is the first evidence that *Gamma*-PVs are also capable of infecting this anatomical site. The role of HPVs in the formation of these lesions has not yet been defined. At least some of these tumors are associated with high-risk HPV infections, particularly with HPV16, which was detected in 22% (8/36) of the samples tested [40]. In another study, which investigated the association between conjunctival SCC formation and *Beta*-PV infection, these viruses were detected in 58% (11/19) of the tumors [41]. In addition, we previously demonstrated that *Beta*-PVs can also be found in verrucous carcinoma (a histological subtype of SCC) of the oral cavity and larynx [31]. In our study, the HPV135 viral load in one of the SCC conjunctival samples was estimated at 72 viral copies/10^4^ cells, which is consistent with the viral load of *Beta*-PVs in cutaneous SCC [38], whereas the viral load was higher in the second HPV135-positive conjunctival SCC (9,900 viral copies/10^4^ cells). Therefore, the question arises as to whether and how *Beta*- and *Gamma*-PVs are involved in the development of malignant tumors of the mucosa, warranting further research.

HPV146 was detected in 13 out of 874 samples. Because the HPV146 viral load in the common wart tissue sample was low (1,134 viral copies/10^4^ cells), and no common causative agents of these neoplasms were identified, it could be suggested that this lesion was caused by a still-unknown HPV type. However, it should be noted that the low viral load in this and our other archival specimens could also be associated with the type of the mentioned samples. Namely, as is well known, the DNA in archival, formalin-fixed, paraffin-embedded (FFPE) tissue samples is prone to degradation, possibly leading to the lower sensitivity of target detection (in our case, viruses) and underestimation of their viral loads.

Except in the tissue sample of the anogential wart, in which the HPV146 viral load was estimated at approximately two copies per cell, the viral load was low in all other HPV146-positive clinical samples (247 to 1,146 viral copies/10^4^ cells). The HPV146-positive anogenital wart also contained HPV6, which is the most common etiological agent of such lesions. Interestingly, in a study of concomitant HPV infections in anogenital warts using laser capture microdissection (LCM) in combination with RT-PCR, only HPV types that were detected exclusively in the lower layers of the infected epithelium (*stratum spinosum* and *stratum basale*) were characterized as their causative agents [42]. Consequently, apart from HPV6 and HPV11, all other HPVs, most commonly high-risk *Alpha*-PVs, were identified as surface contaminants because they were detected only in wart surface smears or in the upper layers of the epithelium [42]. The role of HPV146 in the formation of this anogenital wart could thus be determined only by LCM in combination with RT-PCR or by *in situ* hybridization.

Similarly to HPV135, HPV146 was also detected in anal canal swabs from men who have sex with men, in addition to at least one *Alpha*-PV type; these are commonly associated with the development of anogenital warts. Interestingly, HPV179 infections were not detected in samples from the anogenital area (anogenital warts and cervical and anal swabs), suggesting that the ability of HPV179 to infect the mucosa of this part of the body should be investigated further.

Based on the low HPV146 viral load in most clinical samples tested, it could be concluded that HPV146, at least in immunocompetent individuals, mostly causes clinically insignificant viral infections of the skin and mucosa. In our study, HPV146—which has so far been identified in samples from the oral and nasal cavities, clinically normal skin, and the anogenital area of men and women [4,6,13,15,Chen et al. unpublished data]—was also found in tissue samples of common warts, cutaneous SCC, and the anal canal.

Of note, even though HPVs from the *Beta*- and *Gamma*-PV genera were first described in cutaneous tissues and later also in several mucosal and mucocutaneous samples, it is important to acknowledge that in our study, similarly as in the majority of previously published studies, only PCR-based methods were used for the detection of HPV infections, preventing us from definitely determining whether the infections found in mucosal anatomical sites are indeed mucosal/mucocutaneous or they have originated from the skin, as viral contaminants. The questionable topic could be resolved using the *in-situ* hybridization method or as presented in a study of Hawkins et al. [2013; 42] by a combination of LCM and RT-PCR, which were unfortunately not available to us. Moreover, another limitation preventing us from researching the mentioned question in mucosal tissues is the type of HPV179/135/146-positive samples. Namely, HPV179/135/146 were mostly found in swab samples of the oral cavity, nasopharynx and anal canal and only HPV135 was detected in conjunctival FFPE specimens. Taken together, based on our methods, we assumed that infections, which were found in high viral loads, were most probably not contaminations from other anatomical sites and originated from the site at which they were identified.

In this study, a total of 54 isolates of HPV179 (n = 24), HPV135 (*n* = 17), and HPV146 (*n* = 13) were identified. Given that the majority of clinical specimens were archival formalin-fixed paraffin-embedded tissue samples and surface swabs with low viral loads and/or fragmented viral DNA, the determination of the genetic diversity of *Gamma*-15 PVs was focused solely on the most diverse region of the HPV genome: the non-coding LCR region. In addition, several highly sensitive (at least 10 viral copies/reaction) nested PCR protocols were designed and developed for amplification of the targeted regions. Nevertheless, complete LCRs could not be obtained from all the viral isolates included in the study.

HPV179 isolates obtained from the original immunocompromised patient did not differ within the LCR genomic region, suggesting that the patient had a generalized HPV infection and confirming the results of HPV typing in his samples. Among the other five HPV179 isolates, a single LCR genetic variant was detected, which differed from the reference nucleotide sequence (HG421739) at five nucleotide sites. With the exception of mutations within one potential polyadenylation site for late viral mRNAs (nt site 7,073), all mutations in the HPV179 LCR were detected outside its functional domains, suggesting that HPV179 variants do not differ pathogenically. We hypothesize that, by sequencing a larger number of viral isolates, greater genetic diversity of HPV179 could most probably be demonstrated because according to the previous comparison of all publicly available partial nucleotide sequences of the L1 gene it was suggested that there are many genetic variants and subtypes within HPV types from *Beta*- and *Gamma*-PV genera [43].

All nucleotide sequences of the HPV179 LCR region, including those derived from the immunocompromised patient, differed from the reference HPV179 nucleotide sequence by an insert of a single nucleotide (nt site 7,174). Because the HPV179 reference nucleotide sequence was obtained from the fresh tissue sample, it can be hypothesized that this difference was most probably due to the error of the polymerase during the cloning of the viral genome. According to data from the literature, such events are common in the cloning of PV genomes, especially in sequences rich in repeats of individual nucleotides, mostly AT and GT bp [44–46]. One of the best examples is the reference sequence of HPV6, where a 94 bp long segment of the LCR region was lost during cloning and subsequently identified upon re-sequencing of the original viral isolate [45].

Among the 10 selected HPV135 isolates, four LCR genetic variants were identified and differed from the reference nucleotide sequence (HM999987) by one to six nucleotide substitutions. With the exception of mutations within one potential polyadenylation site for late viral proteins (nt site 6,933), all HPV135 LCR mutations were detected outside its functional domains, suggesting that HPV135 variants do not differ pathogenically. The fact that HPV135 LCR viral variants were detected in samples from different anatomical regions suggests that they are not associated with a specific clinical picture or anatomical site of the infection. However, due to the low number of samples included, this finding warrants further research.

In total, four HPV146 LCR genetic variants were identified among seven HPV146 isolates, differing from the reference sequence (HM999998) by one to eight nucleotide substitutions. According to our results, HPV146 is the most genetically diverse virus among members of the *Gamma*-15 species because eight nucleotide substitutions were demonstrated in as few as two LCR variants. All HPV146 mutations were identified outside important functional LCR domains, suggesting that these viral variants do not differ pathogenically. Because HPV146 mutations have been demonstrated in samples originating from different parts of the body, it could be concluded that they are not associated with a specific clinical picture or anatomical site of infection. However, due to a limited number of samples included in this study, further research is warranted in order to confirm the suggested conclusions.

## 5. Conclusions

In conclusion, HPV179, HPV135 and HPV146 have a mucocutaneous tissue tropism and are associated with sporadic infections in immunocompromised (e.g. after renal transplantation) and immunocompetent individuals. Because the majority of mutations were found outside the major functional domains of the respective LCRs, we assume that HPV179, HPV135, and HPV146 genomic variants pathogenically do not differ from their prototypes. In addition, no association was found between specific HPV179, HPV135, and HPV146 genetic variants and anatomical sites of infection and/or development of specific neoplasms.
